# Airway obstruction due to sticky rice cake (mochi): a case series and review of the literature

**DOI:** 10.1186/s12245-018-0194-7

**Published:** 2018-08-22

**Authors:** Shimpei Nagata, Sung-Ho Kim, Yasuaki Mizushima, Tatsuya Norii

**Affiliations:** 1 Department of Emergency Medicine, Osaka Police Hospital, 10-31 Kitayama-cho, Tennoji-ku, Osaka city, Osaka 543-0035 Japan; 2 Department of Emergency Medicine, University of New Mexico, Albuquerque, USA

**Keywords:** Foreign body airway obstruction, Aspiration, Mochi

## Abstract

**Background:**

Foreign body airway obstruction is a significant public health issue around the world. Mochi, a traditional sticky rice cake in Japan, has gained popularity in many countries including the USA. However, the associated aspiration danger has not yet been well recognized.

**Case presentation:**

We describe three cases of foreign body airway obstruction due to mochi. Case 1 was an elderly man who was brought to the emergency department by an ambulance after he choked on mochi. Despite extensive efforts to remove pieces of mochi including use of Magill forceps, bronchoscopy, and endotracheal intubation, he suffered severe hypoxia and died. Case 2 was a middle-aged man who was found unconscious in a park. The rhythm upon arrival was pulseless electrical activity. During intubation, large pieces of mochi were found in the oropharynx and removed with Magill forceps. He developed aspiration pneumonitis and hypoxic brain injury. The patient was discharged to a skilled nursing facility with severe neurological disability. Case 3 was an elderly man who choked while eating soup with mochi at home. His initial cardiac rhythm was asystole. During intubation, obvious foreign body was found in the oropharynx. Several pieces of mochi were removed by suctioning through the endotracheal tube. He suffered severe hypoxic injury and died.

**Conclusions:**

All of our cases resulted in death or poor neurological outcome. As the popularity of mochi continues to increase, it is likely that cases of aspiration from mochi will also increase. Emergency physician should be aware of the potential danger of mochi and be familiar with the techniques to remove mochi from the airway.

## Background

Foreign body airway obstruction (FBAO) is a significant public health issue around the world. In the US alone, thousands of people die due to FBAO every year [1]. It is a particular problem for both pediatric and geriatric populations. Food is often aspirated in the geriatric population due to inadequate chewing or swallowing dysfunction.

A variety of foods has been reported to cause foreign body aspiration [2, 3]. In Japan, mochi, a traditional sticky rice cake, has received recognition as a significant cause of fatal FBAO due to its unique texture and stickiness.

Globalization of food makes mochi available in many countries outside of Japan. In the USA, the popularity of mochi products has increased and it is commonly available at supermarket chains, particularly as mochi-covered ice cream [4, 5]. However, the associated aspiration danger has not yet been recognized. Awareness of the dangers of this food should promote the development of preventive and treatment strategies.

We report three cases of FBAO due to mochi and suggest preventive and treatment approaches.

## Case presentation

### Case 1

**Fig. 1:**
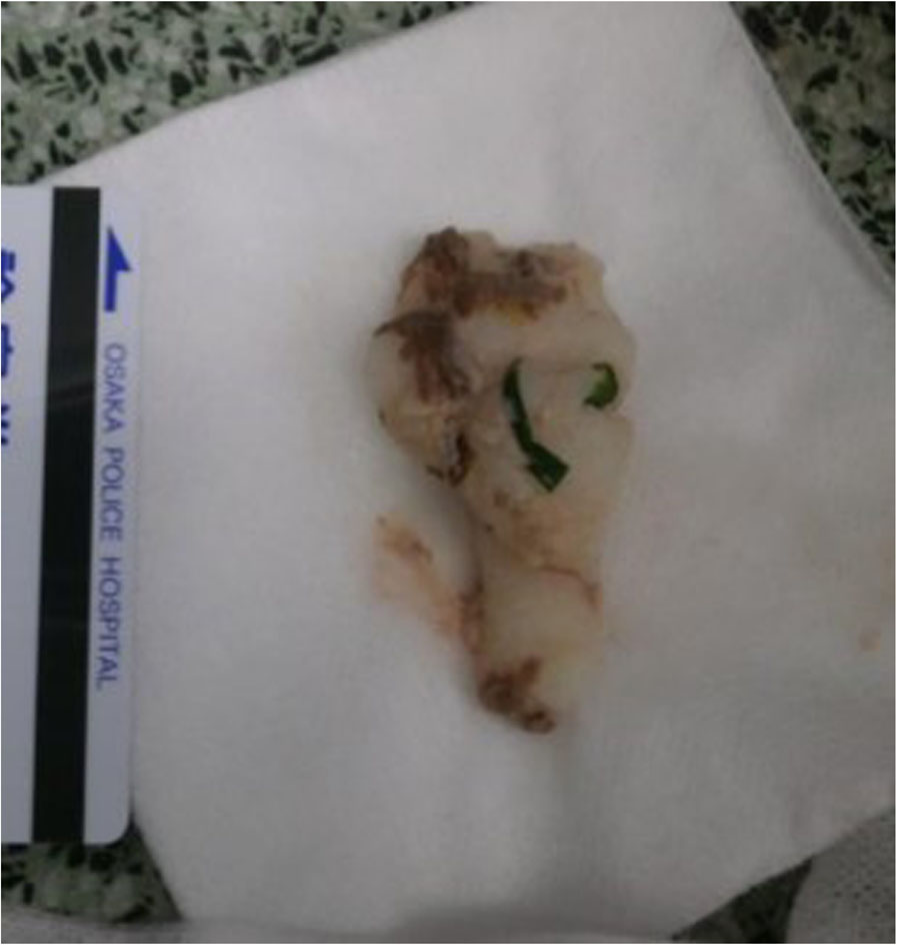
A piece of mochi (65 × 20 mm) removed from the top of the glottis

**Fig. 2:**
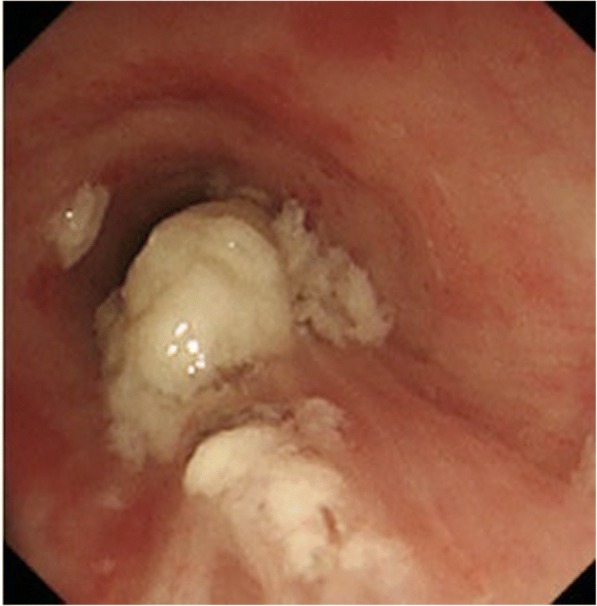
Several large pieces of mochi in the upper part of the trachea

An elderly man with a history of hypertension, who was otherwise healthy, choked on mochi while eating at home. His family attempted to clear mochi from his mouth prior to the arrival of emergency medicine service (EMS). Upon arrival of the patient to the emergency department (ED), his SpO2 was 70% with breathing at 10 L/min of oxygen by a reservoir mask. Due to his increasing agitation and worsening respiratory status, endotracheal intubation was performed. A piece of mochi was found on top of his glottis and removed with a Magill forceps (Fig. 1). After intubation, he continued to have decreased lung sounds on the left side. Bronchoscopy revealed several pieces of mochi in both main bronchi (Fig. 2). Because the size of the fragments was larger than the diameter of intubation tube, they had to be removed by holding the pieces with the biopsy forceps and removing them together with the endotracheal tube. He was then successfully re-intubated without complication. However, he suffered persistent severe respiratory failure due to aspiration pneumonia and died in the intensive care unit 32 days after admission.

### Case 2

**Fig. 3:**
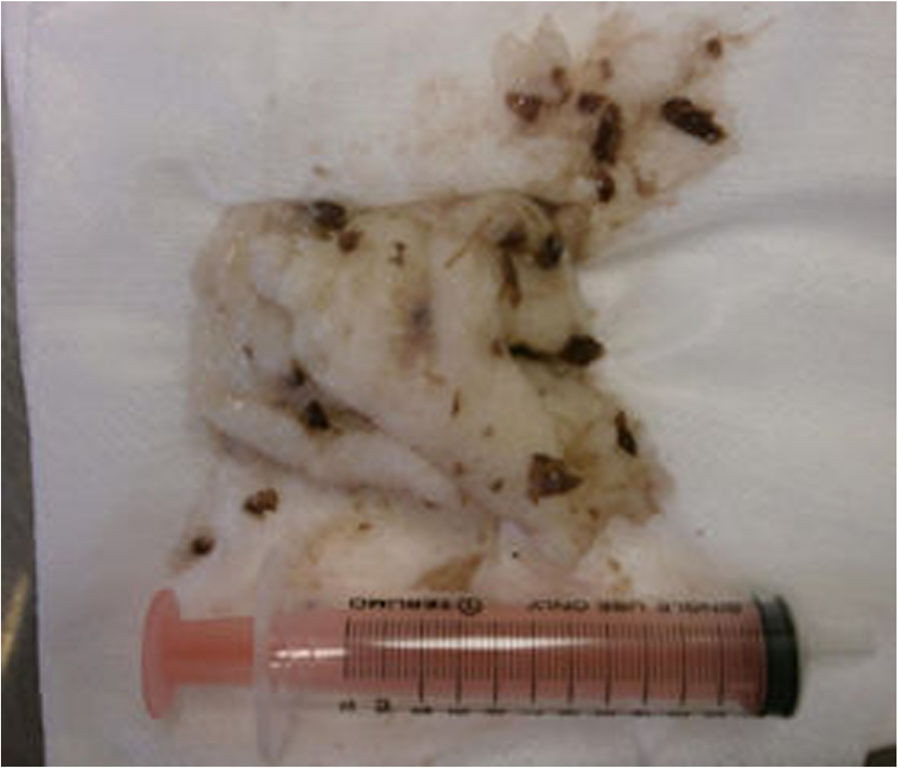
A large piece of mochi (90 × 90 mm) removed from the oropharynx

A middle-aged man was found unconscious and not breathing in a park. Bystander cardiopulmonary resuscitation (CPR) was initiated. No further information about the circumstances of this event was available. He was intubated in the field with a King LTS-D™ supraglottic airway. Upon the arrival of the patient to the ED, CPR was still in progress. The arrival rhythm was pulseless electrical activity (PEA). His lung sounds were diminished in both sides. Upon the exchange of the supraglottic airway device for an endotracheal tube, large pieces of mochi were found in the oropharynx. These pieces of mochi were removed with a Magill forceps (Fig. 3). After which, the patient had return of spontaneous circulation. In the intensive care unit, the patient developed aspiration pneumonitis and hypoxic brain injury. The patient was discharged to a skilled nursing facility on the 19th hospital day. At the time of discharge from our hospital, he had severe neurological disability.

### Case 3

An elderly man with a history of a cerebrovascular accident developed signs of choking while eating soup with mochi at home. Upon arrival of the EMS, he was pulseless and asystole. Advanced cardiac life support was initiated by EMS, and the patient was intubated with King LTS-D ™ supraglottic airway device. Upon the exchange of the supraglottic airway device for an endotracheal tube, several pieces of mochi were removed by aggressive suctioning through the endotracheal tube. After the removal of mochi pieces, the patient had return of spontaneous circulation. However, he died 5 days after admission.

## Conclusions

Mochi, also known as sticky rice cake, is a traditional Japanese food, which is popular in Japan among men and women in all age groups. In the process of making mochi from glutinous rice, external force is applied to make it more cohesive. Similar rice products are widely consumed throughout the east and southeast Asian countries. To our knowledge, there were no cases of FBAO due to mochi in the English literature and only a few articles in the Japanese literature [6–9].

Any upper airway obstruction is potentially life threatening. A variety of conditions including infection, neck trauma, and tumor can cause upper airway obstructions in children and adults [10–12]. Among them, a foreign body, particularly food, is a common cause of airway obstruction [1].

Culture, language, and availability of particular foods affect the type of foods aspirated [3, 13]. Nearly 50 years ago, Haugen et al. described sudden death from FBAO due to incompletely chewed meat as “café coronary syndrome” [2]. This syndrome is typically observed in the elderly with swallowing dysfunction or dentition issues [14]. In contrast to meat as a cause of FBAO in the USA, mochi has been increasing recognized as a potential hazard in the geriatric population in Japan.

The Tokyo Fire Department recently announced that between 2011 and 2015, 562 people were transferred to the ED due to FBAO in Tokyo area and a significant proportion of these cases were caused by mochi aspiration [15]. They warned of the risk of mochi aspiration, particularly in the elderly. The Japanese Consumer Affairs Agency also recently released information about choking from food products, particularly mochi [16].

Food products containing mochi have recently become available in many places outside of Japan [4, 5]. As the popularity of mochi continues to increase, it is likely that cases of FBAO from mochi will also increase. Awareness of the risks associated with mochi and its unique features can have public health and clinical implications in countries outside of Japan.

All of our cases resulted in death or poor neurological outcome, consistent with previous case reports and an epidemiologic study [6, 17]. Due to the lack of controlled research studies comparing types of food among FBAO cases, it is difficult to conclude that the outcome of our mochi cases was particularly worse than other types of food. One of unique risks of mochi is its sticky texture and hardness. When mochi is eaten, it is soft, adheres easily, and chewy at a temperature in a range of 50–60 °C [18]. Later, mochi becomes harder and even more adherent to the larynx and trachea as it cools, and at that stage, mochi is very difficult to remove. Sanpei et al. [19] have proposed replacing waxy wheat mochi as a food alternative to waxy rice mochi because it is less cohesive and adherent. However, it has not been popular in Japan.

A variety of techniques has been described to remove mochi from airways. Similar to other upper airway foreign bodies, if a large piece of mochi is found in the oropharynx or larynx, a Magill forceps can be used for removal. Biopsy forceps, alligator forceps, and basket catheters have also been used to remove mochi pieces found in lower airways [7, 8]. Ueda et al. [9] described the use of a fogarty balloon catheter when other standard techniques failed. Regardless of the technique used, because of its physiochemical characteristics and the urgency of the situation, removal is often challenging. Future studies using a manikin or animal models should be conducted to develop better techniques for removal of mochi from airways. In addition to the techniques described above, extracorporeal membrane oxygenation (ECMO) might be considered for selected cases. Brown et al. [20] reported a successful use of venoarterial ECMO for a 14-month-old boy who suffered from cardiac arrest due to grape aspiration and had full neurological recovery. However, ECMO is not available in many places and requires significant resources [21].

Although extraglottic devices (EGD) for emergency airway management are reasonable choices for many out-of-hospital settings, emergency personnel should be aware of their limitations and FBAO. In our case series, EGDs were placed by EMS in two of three cases. In both cases, the patients were pulseless upon the arrival of EMS at the scene. Because cardiac arrest from FBAO is not uncommon in geriatric population [22, 23], consideration should be given to direct visualization to exclude this possibility if trained personnel are available.

Removal of mochi from the airway is extremely challenging, and all of the three cases had poor outcome. Emergency physicians should be aware of the potential danger of mochi and be familiar with the techniques to remove mochi from the airway. As the popularity of mochi continues to increase, it is likely that cases of aspiration from mochi will also increase. Awareness of the dangers associated with eating mochi can have public health and clinical implications. Public education, warning labels on mochi food products and preparation for appropriate treatment in the ED need to be considered as part of a comprehensive approach to FABO involving mochi.

## Abbreviations

CPR: Cardiopulmonary resuscitation
ED: Emergency department
EGD: Extraglottic devices
EMS: Emergency medicine service
FBAO: Foreign body airway obstruction
PEA: Pulseless electrical activity
